# Supplementary material for: Limitations to photosynthesis by proton motive force-induced photosystem II photodamage
Source: eLife. 2016 Oct 4;5:e16921. doi: 10.7554/eLife.16921 (PMC5050024; doi:10.7554/eLife.16921)
Supplement: Supplementary file 1. — (a) Oligonucleotide sequences utilized for adapter ligation mutagenesis. (b) Oligonucleotide sequences utilized for adapter ligation mutagenesis to introduce secondary mutations. (c) Oligonucleotide sequences utilized for splicing by overlap extension PCR. (d) Synthetic gene constructs incorporating multiple ATPC2 mutations into ATPC1. DOI: http://dx.doi.org/10.7554/eLife.16921.040 [file elife-16921-supp1.docx]

**Supplementary file 1a: Oligonucleotide sequences utilized for adapter ligation mutagenesis.**

| Mutation | *minira* # | Adaptor | Oligonucleotide sequences with mutated nucleotide underlined and in bold |
| --- | --- | --- | --- |
| I201V | *minira* 4 | DMP 27 | 5’ GATCTGTGAC**G**TTAATGGAACCTGTGTGGATGCTGCGGAAGATGAGTTTTTCAGGTT 3’ |
|  |  | DMP 28 | 5’ AACCTGAAAAACTCATCTTCCGCAGCATCCACACAGGTTCCATTAA**C**GTCACA 3’ |
| N202K | *minira* 5 | DMP 29 | 5’ GATCTGTGACATTAA**A**GGAACCTGTGTGGATGCTGCGGAAGATGAGTTTTTCAGGTT 3’ |
|  |  | DMP 30 | 5’ AACCTGAAAAACTCATCTTCCGCAGCATCCACACAGGTTCC**T**TTAATGTCACA 3’ |
| A209I | *minira* 6 | DMP 31 | 5’ GATCTGTGACATTAATGGAACCTGTGTGGATGCT**ATC**GAAGATGAGTTTTTCAGGTT 3’ |
|  |  | DMP 32 | 5’ AACCTGAAAAACTCATCTTC**GAT**AGCATCCACACAGGTTCCATTAATGTCACA 3’ |
| F213M | *minira* 7 | DMP 33 | 5’ GATCTGTGACATTAATGGAACCTGTGTGGATGCTGCGGAAGATGAG**A**T**G**TTCAGGTT 3’ |
|  |  | DMP 34 | 5’ AACCTGAA**C**A**T**CTCATCTTCCGCAGCATCCACACAGGTTCCATTAATGTCACA 3’ |

**Supplementary file 1b: Oligonucleotide sequences utilized for adapter ligation mutagenesis to introduce secondary mutations.**

| Mutation | *minira* # | Adaptor | Oligonucleotide sequences with mutated nucleotide underlined and in bold |
| --- | --- | --- | --- |
| P194M | *minira* 2 | DMP 23 | 5’ GTACACAAAGTTTGTCTCTTTGGTCAAATCAGAACCCGTGATCCACACGCTACTGCCTTTATCA**ATG**AAAGGAGA 3’ |
|  |  | DMP 24 | 5’ GATCTCTCCTTT**CAT**TGATAAAGGCAGTAGCGTGTGGATCACGGGTTCTGATTTGACCAAAGAGACAAACTTTGT 3’ |
| E183D | *minira* 1 | DMP 21 | 5’ GTACACAAAGTTTGTCTCTTTGGTCAAATCAGA**T**CCCGTGATCCACACGCTACTGCCTTTATCACCTAAAGGAGA 3’ |
|  |  | DMP 22 | 5’ GATCTCTCCTTTAGGTGATAAAGGCAGTAGCGTGTGGATCACGGG**A**TCTGATTTGACCAAAGAGACAAACTTTGT 3’ |

**Supplementary file 1c: Oligonucleotide sequences utilized for splicing by overlap extension PCR.**

| Mutation | *minira* # | Region | Primer | Primer sequence |
| --- | --- | --- | --- | --- |
| T218S | *minira* 8 | Fragment 1 | DMP49 | 5’ AACTGTCAATTTCCCTTCTTTACTCGTTAACCT 3’ |
|  |  |  | DMP 45 | 5’ TCCTGCAGCCCGGGAACAAAAAAAT 3’ |
|  |  | Fragment 2 | DMP 50 | 5’ TGAGTTTTTCAGGTTAACGAGTAAAGAAGGG 3’ |
|  |  |  | DMP 46 | 5’ GCGGCCGCTCTAGACAAATCAAAC 3’ |
| E220D | *minira* 9 | Fragment 1 | DMP51 | 5’ AACTGTCAATTTCCCGTCTTTTGTCGTTAAC 3’ |
|  |  |  | DMP 45 | 5’ TCCTGCAGCCCGGGAACAAAAAAAT 3’ |
|  |  | Fragment 2 | DMP 52 | 5’ CAGGTTAACGACAAAAGACGGGAAATT 3’ |
|  |  |  | DMP 46 | 5’ GCGGCCGCTCTAGACAAATCAAAC 3’ |
| T224A | *minira* 10 | Fragment 1 | DMP53 | 5’ GTCTCTCTTTCAACTGCCAATTTCCC 3’ |
|  |  |  | DMP 45 | 5’ TCCTGCAGCCCGGGAACAAAAAAAT 3’ |
|  |  | Fragment 2 | DMP 54 | 5’ CGACAAAAGAAGGGAAATTGGCAGTTGA 3’ |
|  |  |  | DMP 46 | 5’ GCGGCCGCTCTAGACAAATCAAAC 3’ |
| E228T | *minira* 11 | Fragment 1 | DMP55 | 5’ TGTTGGTGTCCTAAAAGTCGTTCTTTCAACTGT 3’ |
|  |  |  | DMP 45 | 5’ TCCTGCAGCCCGGGAACAAAAAAAT 3’ |
|  |  | Fragment 2 | DMP 56 | 5’ GAAATTGACAGTTGAAAGAACGACTTTTAGGA 3’ |
|  |  |  | DMP 46 | 5’ GCGGCCGCTCTAGACAAATCAAAC 3’ |
| I201V-  N202K | *minira* 12 | Fragment 1 | DMP57 | 5’ CACACAGGTTCCTTTCACGTCACAGATCTC 3’ |
|  |  |  | DMP 45 | 5’ TCCTGCAGCCCGGGAACAAAAAAAT 3’ |
|  |  | Fragment 2 | DMP 58 | 5’ GAGATCTGTGACGTGAAAGGAACCTGTGTG 3’ |
|  |  |  | DMP46 | 5’ GCGGCCGCTCTAGACAAATCAAAC 3’ |

**Supplementary file 1d: Synthetic gene constructs incorporating multiple ATPC2 mutations into ATPC1.**

| Domain | *minira* # | Synthetic gene fragment sequence with the new domain in bold. Restriction enzyme sites are underlined. |
| --- | --- | --- |
| 194-241 | *minira* 16 | aattaaTGTACACAAAGTTTGTCTCTTTGGTCAAATCAGAACCCGTGATCCACACGCTACTGCCTTTA**TCGATGAAAGGAGAGTCTTGTGATGTGAAAGGTGAGTGTGTTGATGCTATCGAGGATGAGATGTTTAGGCTAACGAGCAAAGATGGGAAGTTAGCTGTGGAAAGGACCAAGCTTGAAGTTGAGAAGCCTGAGATCTCACCGTTGATG**CAATTCGAGCAAGACCCTGTTCAGATTCTTGATGCTTTGTTGCCTCTGTATCTTAACAGTCAGATTCTTAGGGCATTACAGGAGTCATTGGCTAGTGAGCTTGCAGCTAGAATGAGTGCAATGAGTAGTGCTTCGGATAATGCATCGGATCTCAAGAAATCGCTTTCGATGGTGTATAATAGAAAGCGTCAAGCTAAGATTACTGGAGAGATTCTTGAGATTGTTGCTGGAGCTAATGCACAGGTTTGATTTGTCTAGAttaatt |
| 213-228 | *minira* 14 | aattaaTGTACACAAAGTTTGTCTCTTTGGTCAAATCAGAACCCGTGATCCACACGCTACTGCCTTTATCACCTAAAGGAGAGATCTGTGACATTAATGGAACCTGTGTGGATGCTGCGGAAGATGAG**ATGTTTAGGCTAACGAGCAAAGATGGGAAGTTAGCTGTGGAAAGGACC**ACTTTTAGGACACCAACAGCTGATTTCTCGCCGATCTTGCAATTCGAGCAAGACCCTGTTCAGATTCTTGATGCTTTGTTGCCTCTGTATCTTAACAGTCAGATTCTTAGGGCATTACAGGAGTCATTGGCTAGTGAGCTTGCAGCTAGAATGAGTGCAATGAGTAGTGCTTCGGATAATGCATCGGATCTCAAGAAATCGCTTTCGATGGTGTATAATAGAAAGCGTCAAGCTAAGATTACTGGAGAGATTCTTGAGATTGTTGCTGGAGCTAATGCACAGGTTTGATTTGTCTAGAttaatt |
| I198S | *minira* 3 | aattaaTGTACACAAAGTTTGTCTCTTTGGTCAAATCAGAACCCGTGATCCACACGCTACTGCCTTTATCACCTAAAGGAGAGA**G**CTGTGACATTAATGGAACCTGTGTGGATGCTGCGGAAGATGAGTTTTTCAGGTTAACGACAAAAGAAGGGAAATTGACAGTTGAAAGAGAGACTTTTAGGACACCAACAGCTGATTTCTCGCCGATCTTGCAATTCGAGCAAGACCCTGTTCAGATTCTTGATGCTTTGTTGCCTCTGTATCTTAACAGTCAGATTCTTAGGGCATTACAGGAGTCATTGGCTAGTGAGCTTGCAGCTAGAATGAGTGCAATGAGTAGTGCTTCGGATAATGCATCGGATCTCAAGAAATCGCTTTCGATGGTGTATAATAGAAAGCGTCAAGCTAAGATTACTGGAGAGATTCTTGAGATTGTTGCTGGAGCTAATGCACAGGTTTGATTTGTCTAGAttaatt |
