# Supplementary material for: Limitations to photosynthesis by proton motive force-induced photosystem II photodamage
Source: eLife. 2016 Oct 4;5:e16921. doi: 10.7554/eLife.16921 (PMC5050024; doi:10.7554/eLife.16921)
Supplement: Supplementary file 2. — (a) Timing and light profile of imaging day one. (b) Timing and light profile of imaging day two. (c) Timing and light profile of imaging day three. DOI: http://dx.doi.org/10.7554/eLife.16921.041 [file elife-16921-supp2.docx]

**Supplementary file 2a: Timing and light profile of imaging day 1**

| Light Intensity Day 1 | Duration at Intensity (min) | Time of Day |
| --- | --- | --- |
| 0 | 359.4 | 6:00 |
| 100 | 58 | 6:58 |
| 0 | 2 | 6:00 |
| 100 | 58 | 7:58 |
| 0 | 2 | 7:00 |
| 100 | 58 | 8:58 |
| 0 | 2 | 8:00 |
| 100 | 58 | 9:58 |
| 0 | 2 | 9:00 |
| 100 | 58 | 10:58 |
| 0 | 2 | 10:00 |
| 100 | 58 | 11:58 |
| 0 | 2 | 11:00 |
| 100 | 58 | 12:58 |
| 0 | 2 | 12:00 |
| 100 | 58 | 13:58 |
| 0 | 2 | 13:00 |
| 100 | 58 | 14:58 |
| 0 | 2 | 14:00 |
| 100 | 58 | 15:58 |
| 0 | 2 | 15:00 |
| 100 | 58 | 16:58 |
| 0 | 2 | 16:00 |
| 100 | 58 | 17:58 |
| 0 | 2 | 17:00 |
| 100 | 58 | 18:58 |
| 0 | 2 | 18:00 |
| 100 | 58 | 19:58 |
| 0 | 2 | 19:00 |
| 100 | 58 | 20:58 |
| 0 | 2 | 20:00 |
| 100 | 58 | 21:58 |
| 0 | 2 | 22:00 |

**Supplementary file 2b: Timing and light profile of imaging day 2**

| Light Intensity Day 2 | Duration at Intensity (min) | Time of Day |
| --- | --- | --- |
| 0 | 359.4 | 6:00 |
| 39 | 28 | 6:28 |
| 0 | 2 | 6:30 |
| 80 | 28 | 6:58 |
| 0 | 2 | 7:00 |
| 123 | 28 | 7:28 |
| 0 | 2 | 7:30 |
| 167 | 28 | 7:58 |
| 0 | 2 | 8:00 |
| 210 | 28 | 8:28 |
| 0 | 2 | 8:30 |
| 253 | 28 | 8:58 |
| 0 | 2 | 9:00 |
| 294 | 28 | 9:28 |
| 0 | 2 | 9:30 |
| 333 | 28 | 9:58 |
| 0 | 2 | 10:00 |
| 370 | 28 | 10:28 |
| 0 | 2 | 10:30 |
| 402 | 28 | 10:58 |
| 0 | 2 | 11:00 |
| 431 | 28 | 11:28 |
| 0 | 2 | 11:30 |
| 455 | 28 | 11:58 |
| 0 | 2 | 12:00 |
| 475 | 28 | 12:28 |
| 0 | 2 | 12:30 |
| 489 | 28 | 12:58 |
| 0 | 2 | 13:00 |
| 497 | 28 | 13:28 |
| 0 | 2 | 13:30 |
| 500 | 28 | 13:58 |
| 0 | 2 | 14:00 |
| 500 | 28 | 14:28 |
| 0 | 2 | 14:30 |
| 497 | 28 | 14:58 |
| 0 | 2 | 15:00 |
| 489 | 28 | 15:28 |
| 0 | 2 | 15:30 |
| 475 | 28 | 15:58 |
| 0 | 2 | 16:00 |
| 455 | 28 | 16:28 |
| 0 | 2 | 16:30 |
| 431 | 28 | 16:58 |
| 0 | 2 | 17:00 |
| 402 | 28 | 17:28 |
| 0 | 2 | 17:30 |
| 370 | 28 | 17:58 |
| 0 | 2 | 18:00 |
| 333 | 28 | 18:28 |
| 0 | 2 | 18:30 |
| 294 | 28 | 18:58 |
| 0 | 2 | 19:00 |
| 253 | 28 | 19:28 |
| 0 | 2 | 19:30 |
| 210 | 28 | 19:58 |
| 0 | 2 | 20:00 |
| 167 | 28 | 20:28 |
| 0 | 2 | 20:30 |
| 123 | 28 | 20:58 |
| 0 | 2 | 21:00 |
| 80 | 28 | 21:28 |
| 0 | 2 | 21:30 |
| 39 | 28 | 21:58 |
| 0 | 2 | 22:00 |
|  |  |  |

**Supplementary file 2c: Timing and light profile of imaging day 3**

| Light Intensity Day 3 | Duration at Intensity (min) | Time of Day |
| --- | --- | --- |
| 0 | 359.4 | 6:00 |
| 39 | 18 | 6:18 |
| 0 | 2 | 6:20 |
| 78 | 8 | 6:28 |
| 0 | 2 | 6:30 |
| 80 | 18 | 6:48 |
| 0 | 2 | 6:50 |
| 161 | 8 | 6:58 |
| 0 | 2 | 7:00 |
| 123 | 18 | 7:18 |
| 0 | 2 | 7:20 |
| 246 | 8 | 7:28 |
| 0 | 2 | 7:30 |
| 167 | 18 | 7:48 |
| 0 | 2 | 7:50 |
| 333 | 8 | 7:58 |
| 0 | 2 | 8:00 |
| 210 | 18 | 8:18 |
| 0 | 2 | 8:20 |
| 420 | 8 | 8:28 |
| 0 | 2 | 8:30 |
| 253 | 18 | 8:48 |
| 0 | 2 | 8:50 |
| 506 | 8 | 8:58 |
| 0 | 2 | 9:00 |
| 294 | 18 | 9:18 |
| 0 | 2 | 9:20 |
| 588 | 8 | 9:28 |
| 0 | 2 | 9:30 |
| 333 | 18 | 9:48 |
| 0 | 2 | 9:50 |
| 667 | 8 | 9:58 |
| 0 | 2 | 10:00 |
| 370 | 18 | 10:18 |
| 0 | 2 | 10:20 |
| 739 | 8 | 10:28 |
| 0 | 2 | 10:30 |
| 402 | 18 | 10:48 |
| 0 | 2 | 10:50 |
| 805 | 8 | 10:58 |
| 0 | 2 | 11:00 |
| 431 | 18 | 11:18 |
| 0 | 2 | 11:20 |
| 862 | 8 | 11:28 |
| 0 | 2 | 11:30 |
| 455 | 18 | 11:48 |
| 0 | 2 | 11:50 |
| 911 | 8 | 11:58 |
| 0 | 2 | 12:00 |
| 475 | 18 | 12:18 |
| 0 | 2 | 12:20 |
| 949 | 8 | 12:28 |
| 0 | 2 | 12:30 |
| 489 | 18 | 12:48 |
| 0 | 2 | 12:50 |
| 977 | 8 | 12:58 |
| 0 | 2 | 13:00 |
| 497 | 18 | 13:18 |
| 0 | 2 | 13:20 |
| 994 | 8 | 13:28 |
| 0 | 2 | 13:30 |
| 500 | 18 | 13:48 |
| 0 | 2 | 13:50 |
| 1000 | 8 | 13:58 |
| 0 | 2 | 14:00 |
| 500 | 18 | 14:18 |
| 0 | 2 | 14:20 |
| 1000 | 8 | 14:28 |
| 0 | 2 | 14:30 |
| 497 | 18 | 14:48 |
| 0 | 2 | 14:50 |
| 994 | 8 | 14:58 |
| 0 | 2 | 15:00 |
| 489 | 18 | 15:18 |
| 0 | 2 | 15:20 |
| 977 | 8 | 15:28 |
| 0 | 2 | 15:30 |
| 475 | 18 | 15:48 |
| 0 | 2 | 15:50 |
| 949 | 8 | 15:58 |
| 0 | 2 | 16:00 |
| 455 | 18 | 16:18 |
| 0 | 2 | 16:20 |
| 911 | 8 | 16:28 |
| 0 | 2 | 16:30 |
| 431 | 18 | 16:48 |
| 0 | 2 | 16:50 |
| 862 | 8 | 16:58 |
| 0 | 2 | 17:00 |
| 402 | 18 | 17:18 |
| 0 | 2 | 17:20 |
| 805 | 8 | 17:28 |
| 0 | 2 | 17:30 |
| 370 | 18 | 17:48 |
| 0 | 2 | 17:50 |
| 739 | 8 | 17:58 |
| 0 | 2 | 18:00 |
| 333 | 18 | 18:18 |
| 0 | 2 | 18:20 |
| 667 | 8 | 18:28 |
| 0 | 2 | 18:30 |
| 294 | 18 | 18:48 |
| 0 | 2 | 18:50 |
| 588 | 8 | 18:58 |
| 0 | 2 | 19:00 |
| 253 | 18 | 19:18 |
| 0 | 2 | 19:20 |
| 506 | 8 | 19:28 |
| 0 | 2 | 19:30 |
| 210 | 18 | 19:48 |
| 0 | 2 | 19:50 |
| 420 | 8 | 19:58 |
| 0 | 2 | 20:00 |
| 167 | 18 | 20:18 |
| 0 | 2 | 20:20 |
| 333 | 8 | 20:28 |
| 0 | 2 | 20:30 |
| 123 | 18 | 20:48 |
| 0 | 2 | 20:50 |
| 246 | 8 | 20:58 |
| 0 | 2 | 21:00 |
| 80 | 18 | 21:18 |
| 0 | 2 | 21:20 |
| 161 | 8 | 21:28 |
| 0 | 2 | 21:30 |
| 39 | 18 | 21:48 |
| 0 | 2 | 21:50 |
| 78 | 8 | 21:58 |
| 0 | 2 | 22:00 |
