# Supplementary material for: Limitations to photosynthesis by proton motive force-induced photosystem II photodamage
Source: eLife. 2016 Oct 4;5:e16921. doi: 10.7554/eLife.16921 (PMC5050024; doi:10.7554/eLife.16921)
Supplement: Supplementary file 3. — All measurements were performed on three-week old leaves following in vivo spectroscopic measurements as described in Materials and methods. Data represent the mean ± s.d. for n ≥ 3 leaves. Statistically significant differences (*p<0.05) from wild-type were determined using a t-test. DOI: http://dx.doi.org/10.7554/eLife.16921.042 [file elife-16921-supp3.docx]

**Supplementary file 3: Chlorophyll content of wild-type (Ws-2) and *minira* leaves.**

| Genotype | Chlorophyll (μg cm^-2^) |
| --- | --- |
| Ws-2 | 16.55 + 0.51 |
| *minira* 2-2 | 13.00 + 0.17^*^ |
| *minira* 3-1 | 8.24 + 1.62^*^ |
| *minira* 3-2 | 13.04 + 1.45 |
| *minira* 4-1 | 14.55 + 1.18 |
| *minira* 4-2 | 14.05 + 1.34 |
| *minira* 4-3 | 14.99 + 0.78 |
| *minira* 6-1 | 13.64 + 0.78^*^ |
| *minira* 6-2 | 13.74 + 0.85^*^ |
| *minira* 7-1 | 13.48 + 0.25^*^ |
| *minira* 8-1 | 10.51 + 1.53^*^ |
| *minira* 9-1 | 12.53 + 2.85 |
| *minira* 11-1 | 11.98 + 1.52^*^ |
| *minira* 12-2 | 8.46 + 2.28^*^ |
| *minira* 12-3 | 12.28 + 0.94^*^ |
| *minira* 14-1 | 13.87 + 1.65 |
